# A high throughput and micrometre spatial resolution design for a versatile resonant inelastic X-ray scattering spectrometer

**DOI:** 10.1107/S1600577525005314

**Published:** 2025-07-17

**Authors:** Ruijie Wang, Pengjun Zhang, Qingchen Li, Yujian Xia, Lisheng Qian, Xuefei Feng, Shuangming Chen, Xiaosong Liu

**Affiliations:** a National Synchrotron Radiation Laboratory University of Science and Technology of China Hefei Anhui230029 People’s Republic of China; b Department of Optics and Optical Engineering University of Science and Technology of China Hefei Anhui230026 People’s Republic of China; Advanced Photon Source, USA

**Keywords:** synchrotron radiation, soft X-ray spectrometer, resonant inelastic X-ray scattering spectroscopy

## Abstract

A versatile scheme has been meticulously engineered for a resonant inelastic X-ray scattering spectrometer, enabling free switching between high throughput configurations with narrow spatial-resolution and low throughput setups boasting broad spatial-resolution capabilities. The high throughput mode enhances signal intensity threefold compared with setups with only the dispersive subassembly, while achieving 2.7 µm resolution within a 16 µm range. Alternatively, the broad spatial-resolution mode achieves 2.7 µm resolution across the entire optical footprint. Furthermore, the incorporation of specialized mirrors not only facilitates focusing but also paves the way for potential enhancements to the strip-shaped detector, thereby augmenting its resolution. By employing a vertically compact beam spot in conjunction with the Hettrick–Underwood optical configuration, this spectrometer attains a resolving power surpassing 12000 for 284–780 eV. This work provides an exhaustive exploration of the multifaceted attributes to the spectrometer’s optical design, highlighting its adaptability and high performance in diverse experimental scenarios.

## Abstract 2

A versatile resonant inelastic X-ray scattering spectrometer seamlessly switches between high throughput and broad spatial-resolution modes: (i) the high throughput mode enhances signal intensity threefold while achieving 2.7 µm resolution within a 16 µm range; (ii) the broad spatial-resolution mode achieves entire optical footprint resolution at the expense of throughput. This innovative system, featuring a resolving power exceeding 12000, is poised to enhance experimental capabilities in energy materials research.

## 1. Introduction

Resonant inelastic X-ray scattering (RIXS) has undergone significant development as a spectroscopic technique at synchrotron facilities over the past few decades, such as the high-resolution RIXS spectrometer at Diamond Light Source, spatial-resolved RIXS spectrometer at ALS, and time-resolved RIXS spectrometer at Swiss Light Source (Chuang *et al.*, 2020 ▸; Monney *et al.*, 2023 ▸; Zhou *et al.*, 2022 ▸). RIXS is a photon-in/photon-out spectroscopy, which reflects the electronic structure of matter by comparing the energy of incoming photons and emitted photons when the matter is excited by the incoming photons’ energies tuned to the elemental absorption edges (Lundberg & Wernet, 2020 ▸). Based on this principle, the design of the spectrometer mainly focuses on three aspects. Firstly, high energy resolution enables the RIXS signal to reveal more information. The ongoing pursuit of higher resolution is primarily focused on the study of elementary excitations in quantum materials, such as charge, magnons and phonons (Lee *et al.*, 2021 ▸; Ruiz *et al.*, 2021 ▸). Dedicated spectrometers targeting research in this field typically employ design strategies such as lengthening the spectrometer and optimizing grating configurations in order to achieve an energy resolution of less than 20 meV at 1 keV (De Groot *et al.*, 2024 ▸; Dvorak *et al.*, 2016 ▸; Ghiringhelli *et al.*, 2006 ▸; Hettrick *et al.*, 1988 ▸). Secondly, RIXS detection also aims to unveil chemical valence, spin states, coordination changes, and more in materials for biological and energy applications. While these fields have lower energy resolution requirements than the former, spectrometers need to strive for maximum throughput. And throughput holds even greater importance in spectrometers equipped with *in situ* capabilities due to additional flux loss from the *in situ* device and quick spectrum acquisition process (Liu *et al.*, 2014 ▸; Qiao *et al.*, 2017 ▸). Thirdly, the development of multifunctionality is also a significant goal for the spectrometer. For example, regarding heterogeneous energy materials, significant progress has been made in electronic signal analysis through scanning electron microscopy (SEM) and scanning transmission electron microscopy (STEM) to examine surface morphology (Barroo *et al.*, 2020 ▸; He *et al.*, 2019 ▸). However, the average signal is collected by the entire area of the detector in previous spectrometer designs, unable to be used for the above research. Currently, the development of spatial resolution is also being considered, but no concrete construction or application has been implemented (Chuang *et al.*, 2020 ▸).

In practical design, both high throughput and spatial resolution are seldom achieved simultaneously in a spectrometer. Mainstream methods for achieving spatial resolution are primarily based on using micrometre-sized small spots for multiple tests at different positions on the sample (Flank *et al.*, 2006 ▸). However, this approach is clearly unsuitable for collecting electronic structure information *in situ* or *operando* due to concerns about instantaneity. Alternatively, adding imaging components before the grating is also discussed and may be suitable for *in situ* conditions. However, these approaches can complicate the integration of additional functions within the optical design or machine design; this is not consistent with the design philosophy of high throughput. To enhance throughput, the optical design of a spectrometer typically emphasizes minimizing the number of optical components to reduce reflection losses, as well as incorporating collection mirror systems to increase throughput by eliminating the size limitations of the detector (Chuang *et al.*, 2016 ▸; Dvorak *et al.*, 2016 ▸; Huang & Chen, 2018 ▸; Zhou *et al.*, 2022 ▸). Therefore, building a spectrometer that can simultaneously integrate high throughput and spatial resolution for applications in energy materials remains a significant challenge.

In this work, we present a design for a RIXS spectrometer that integrates high throughput and spatial-resolution operational modes. The spectrometer employs three functional modules: (i) a switching assembly enabling operational mode transitions, (ii) a dispersive subassembly for energy resolution of emitted photons, and (iii) a detector for final data collection. Inspired by existing spectrometer optical designs (Chuang *et al.*, 2020 ▸; Warwick *et al.*, 2014 ▸), the switching assembly is positioned in front of the dispersion assembly, and provides two options for distinct focusing methods in the non-dispersive direction: the horizontal focusing mirror (HFM) and a set of Wolter-type mirrors (WM). Firstly, the specific optical scheme and the essential energy resolution of the spectrometer are detailed in Section 2. Because of the limitations of independent focusing in both directions, the dispersive subassembly of the spectrometer utilizes a VLS-Hettrick–Underwood design (Dvorak *et al.*, 2016 ▸; Hettrick *et al.*, 1988 ▸). Then, in the switching assembly, the HFM is used as a collection mirror to alleviate the constraints of detector size on the collection angle, and to reduce detector noise through the focused small beam spot. Meanwhile, the HFM provides the spectrometer with spatial resolution capabilities over a limited area. The performance evaluation of the HFM is described in Section 3. In order to accommodate a broader range of application prospects, Wolter-type mirrors (WM1 and WM2) are added parallel to the HFM, although this results in a sacrifice of throughput. The specific details regarding these two aspects of the WM scheme are discussed in Section 4. Considering the relatively rare two-dimensional focused spectrometer, Section 5 further evaluates the interactions that occur in achieving simultaneous functionality. Finally, the small beam spot is very sensitive to the position of optical components, which can significantly affect both the energy resolution and spatial resolution of the spectrometer. Therefore, in Section 6, we evaluate the mechanical tolerances of each component intended for the mechanical design.

## 2. Overview of the spectrometer

### 2.1. Design concept of the spectrometer

As an analytical instrument for resolving the energy information of emitted photons from samples, the basic principle of the RIXS spectrometer lies in using dispersive optics (grating) to image the dispersed photons onto a detector. The energy resolution of the scattered signal is achieved through the pixels on the detector. Therefore, both the grating line density and the detector’s pixel size collectively determine the overall energy resolution of the spectrometer. However, since the scattered photons from the sample are distributed across the entire solid angle, the physical dimensions of the optics and detector inherently limit the range of angles that can be collected. To address this, the collection mirror perpendicular to the grating is typically installed upstream of the grating. This mirror extends the collection angle by focusing light in the non-dispersive direction. Furthermore, this position can be replaced with other functional components to extend the spectrometer’s capabilities. Based on the design logic, the spectrometer in this design is composed of three post-sample functional modules: (i) a switching assembly enabling operational mode transitions, (ii) a dispersive subassembly for energy resolution of emitted photons, and (iii) the detector for final data collection. As the key element for energy analysis in the spectrometer, we will first introduce the performance of the dispersive subassembly in this design.

### 2.2. Optical scheme for energy resolution

**Figure 1:**
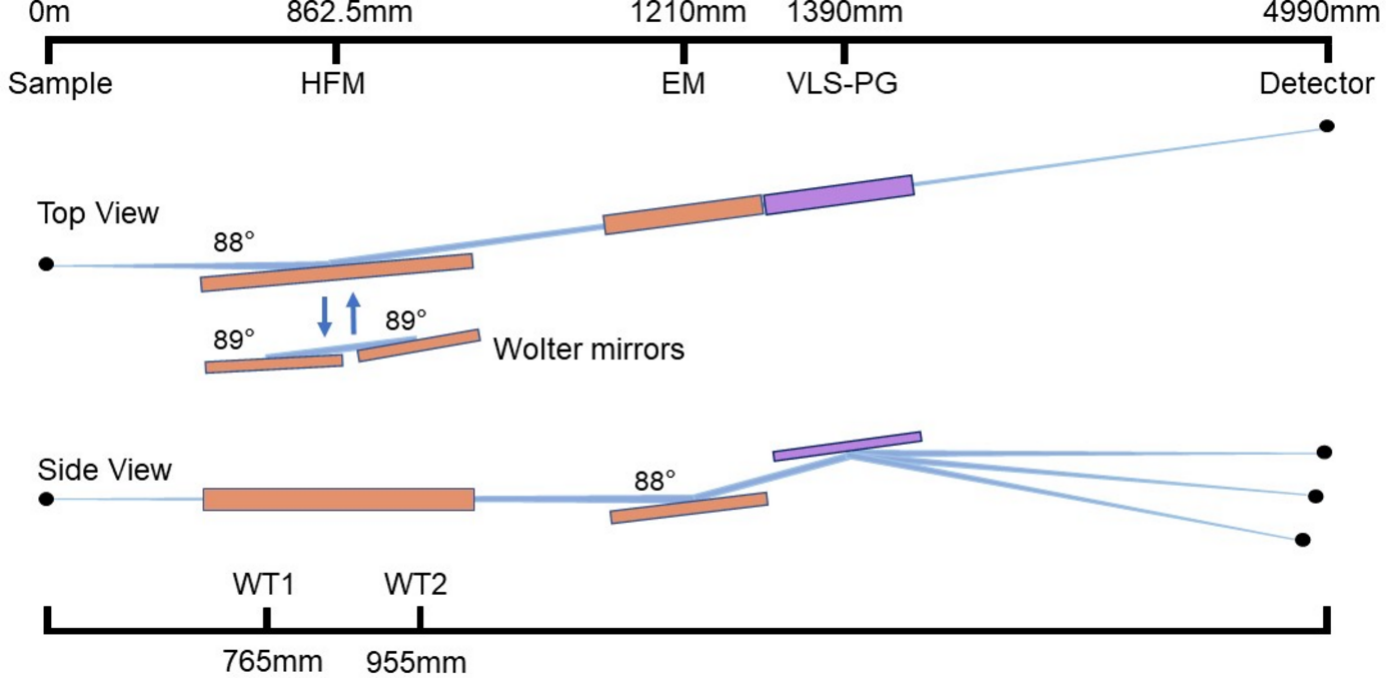
Top and side views of the layout of the spectrometer.

The RIXS spectrometer design takes into account the beam spot size commonly used at other beamlines, with the beam size defined as 80 µm (H) × 5 µm (V). First of all, the constraints of the spectrometer have been summarized. The total length must be controlled within 6 m because of space constraints. A commercial sCMOS detector with a 6.5 µm pixel size and a 13.3 mm total effective size will be used. Meanwhile, the resolving power is expected to reach 12000 at 284 eV (C *K*-edge) and 780 eV (3*d* metal) using two gratings. The optical layout of the spectrometer is illustrated in Fig. 1 ▸.

Due to the emitted photons being focused simultaneously by the HFM and the dispersive subassembly, fixed object and image distances are the limiting factors in the dispersive subassembly design, then the Hettrick Underwood (HU) design is chosen by comparing various schemes (Dvorak *et al.*, 2016 ▸; Hettrick *et al.*, 1988 ▸). The HU design includes two parts: a converging mirror and a plane grating with varied line spacing (VLS) rulings. For the VLS grating, it has a self-focusing function according to (Xue *et al.*, 2019 ▸)
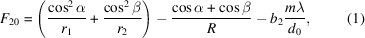
where *F*_20_ is the defocus term, α and β are the incidence and diffraction angles, *r*_1_ and *r*_2_ are the object and image distance, *R* is the grating radius (for a plane grating, *R* → ∞), *m* is the diffraction order of the grating (*m* = +1), and *b*_2_ is the space-variation parameter. When *F*_20_ = 0, the focusing condition is satisfied, then *b*_2_ is calculated by
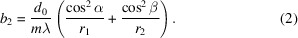
If *r*_1_ = −*r*_2_, *b*_2_ will be approximated as follows (Sinn *et al.*, 2012 ▸),
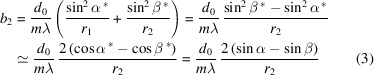
where 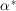 and 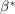 are the complementary angles of α and β. Meanwhile, the grating equation is considered, then *b*_2_ = 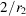 is obtained. As the energy is related to α and β, *b*_2_ is independent of the energy when *r*_1_ = −*r*_2_. Then a converging mirror is used in the HU design to pre-focus; the obtained virtual source makes the object distance equal to the imaging distance of the VLS grating (Li *et al.*, 2024 ▸). In our design, an elliptical cylindrical mirror (EM) was selected as the converging mirror, and two plane gratings were designed to cover the energy range and achieve the required resolving power. The parameters of the optical components are summarized in Table 1 ▸. The impact of the VLS terms’ errors on energy resolution, along with the corresponding correction methods, has been included in Section 2 of the supporting information.

In addition to the grating, the final energy resolution also depends on the image distance (*r*_2_) determined by the detector position. The principle of setting the detector position is that the resolving power of 12000 (energy resolution of ∼65 meV at 780 eV) cannot be limited by the sCMOS detector with 13 µm effective pixel resolution (*Dp*, twice the pixel size). Then *r*_2_ is calculated by
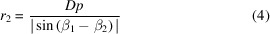
where β_1_ and β_2_ are the diffraction angles at 780 eV and 780.065 eV, respectively, so the calculated result for *r*_2_ is about 3600 mm.

### 2.3. Energy resolution of the spectrometer

**Figure 2:**
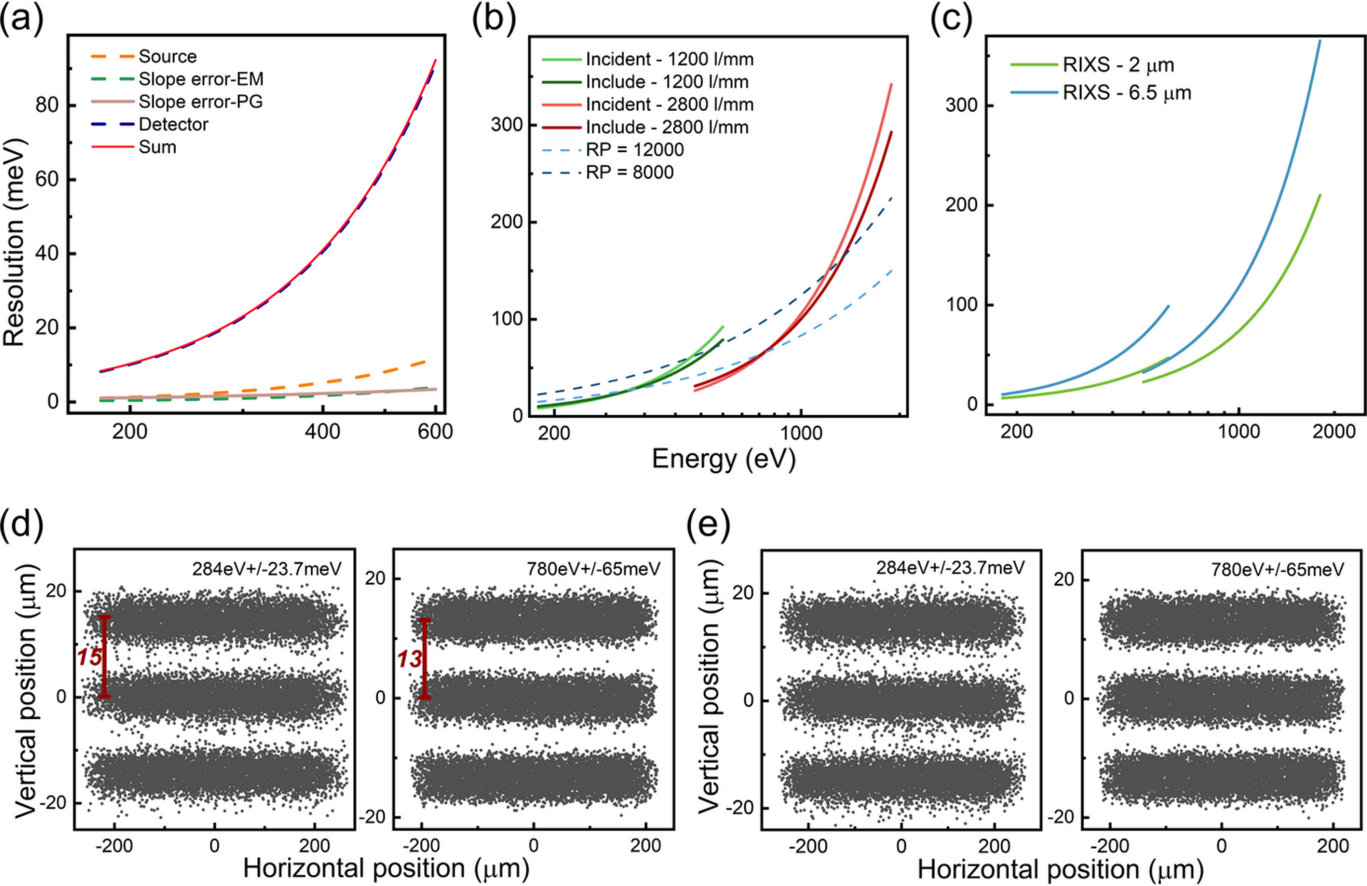
Energy resolution of the spectrometer. (*a*) The contribution of various factors on the calculated resolution under the constant incident angle method within the LEG. (*b*) Total resolution for different methods and gratings. (*c*) Total resolution under different sCMOS settings (2 µm pixel and 6.5 µm pixel). (*d*) *SHADOW* simulations at the detector plane with the constant incident angle method at 284 eV (using the LEG) and 780 eV (using the HEG). (*e*) *SHADOW* simulations at the detector plane with the constant included angle (172°) method at 284 eV (using the LEG) and 780 eV (using the HEG).

**Figure 3:**
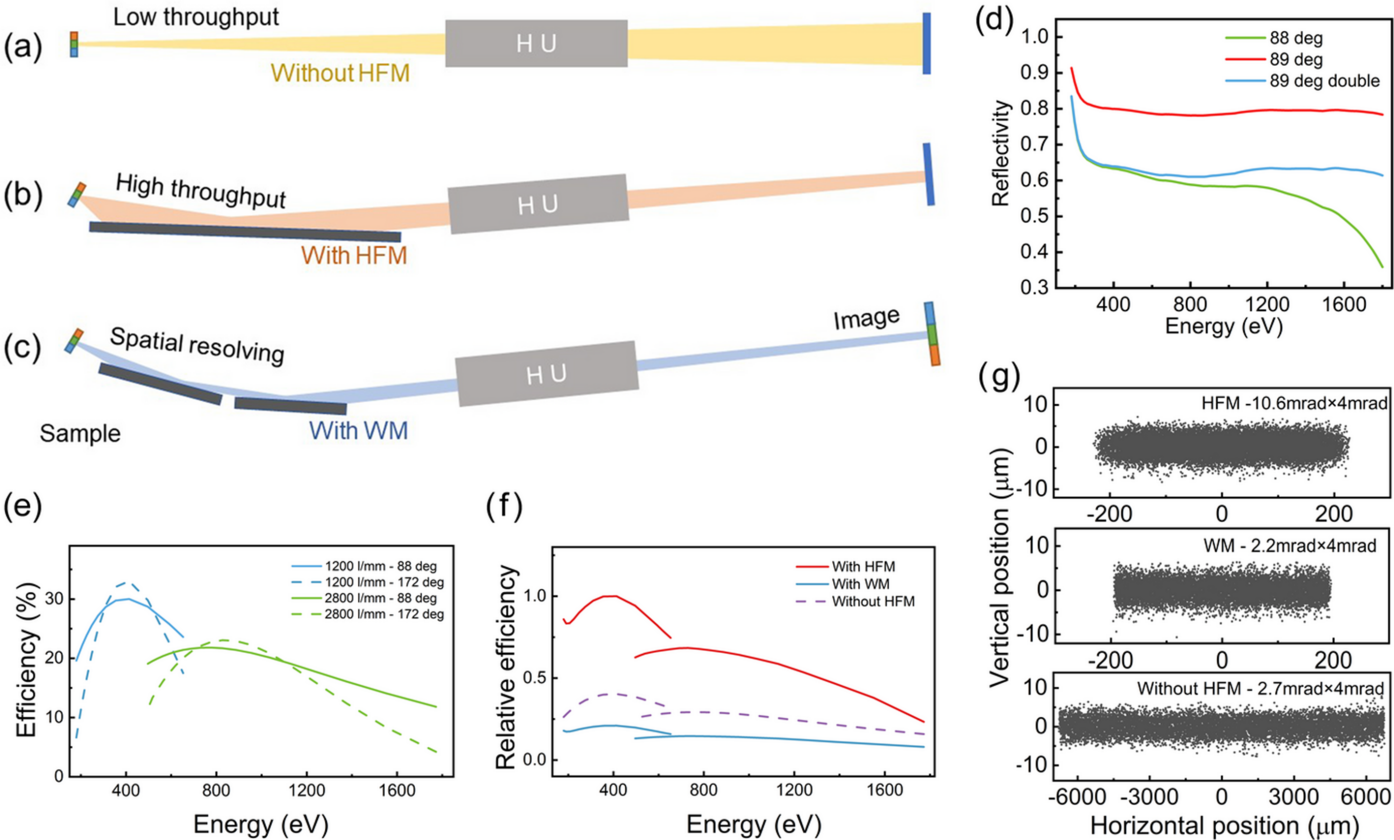
(*a*)–(*c*) Diagrams illustrating the collection angle for the non-collection mirror, HFM and Wolter mirrors schemes. (*d*) The simulated reflectivity of the gold coating as a function of energy with different incident angles (green line and red line), and the square of the reflectivity at 89° (red line) corresponding to two reflections in the Wolter mirrors scheme. (*e*) Simulated LEG and HEG efficiencies for different methods as a function of energy. The solid line represents the constant incident angle method (88°), while the dashed line represents the constant included angle method (172°). (*f*) Total relative efficiencies for different schemes as a function of energy; the grating efficiencies with the constant incident angle method is used to calculate total efficiencies, and the data have been normalized. (*g*) *SHADOW* simulations at the detector plane with different mirror schemes and divergence angle settings.

The dispersive subassembly is equipped with two gratings: the low-energy grating (LEG) and the high-energy grating (HEG), which have line densities of 1200 and 2800 lines mm^−1^, respectively. The energy range covered and VLS parameters are summarized in Table 1 ▸. Operation of the grating at both constant incident angle (α) and constant included angle (2θ) is planned, which will determine two different motion mechanisms for detector movement and grating rotation, respectively. The method of grating rotation will provide users with a simpler experience, as energy adjustment only requires the grating to rotate rather than moving the entire optical tube connected to the detector. The calculated resolution results (Xue *et al.*, 2019 ▸) for two methods are shown in Fig. 2 ▸(*b*). It can be concluded that the resolution of the two methods is fundamentally equivalent. However, a disparity is noted in the diffraction efficiency of the grating between the two methods, as shown in Fig. 3 ▸(*e*). In the high-energy (>1000 eV) and the low-energy (<300 eV) ranges, the constant incident angle method demonstrates higher efficiency, reaching up to twice the amount at its peak. In the range 300–1000 eV, the constant included angle method is more conducive to signal acquisition. Note that, to ensure the universality of the grating for two different motion mechanisms, the incident angle (88°) and included angle (172°) were determined based on the VLS parameters, enabling compatibility with both configurations. Additionally, *Gsolver* (https://gsolver.software.informer.com/) is used to calculate the grating efficiency, and the gratings are configured as blazed gratings, with blaze angles of 1.7° (1200 lines mm^−1^) and 1.9° (2800 lines mm^−1^). The efficiency calculations for the laminar grating are shown in Fig. S4 of the supporting information, demonstrating significantly lower efficiency compared with the blazed grating. Consequently, the blazed grating has been selected as the final solution.

The calculated energy resolution of different contributing factors is shown in Fig. 2 ▸(*a*), taking the constant incident angle method of the LEG as an example. The contribution of the detector almost overlaps with the total resolution curve. It indicates that pixel size is the primary factor limiting the resolution at present. Considering the upgrade of the later detectors, Fig. 2 ▸(*c*) shows the combined resolution of the beamline and spectrometer with 6.5 µm pixel (13 µm effective pixel) or 2 µm pixel (4 µm effective pixel). Different from the results when limited by the detector at the existing 6.5 µm pixel, the resolution of 13.8 meV is achieved at 284 eV with 2 µm pixel (mainly limited by the beamline). In conclusion, there is significant potential for improvement in the energy resolution by development of the sCMOS detector.

The energy resolution of the two methods is simulated using *SHADOW* in Figs. 2 ▸(*d*) and 2 ▸(*e*). Firstly, the divergence at 780 eV (resolving power of 12000) is verified to be approximately 13 µm when the detector is placed 3600 mm away from the grating. This is consistent with the theoretical calculations. Other energies are shown in Figs. S2 and S3.

## 3. Mode 1: horizontal focusing mirrors

Before the dispersive subassembly, the switching assembly enables the spectrometer to operate in two distinct modes: the first is a high throughput mode facilitated by the elliptical cylindrical mirror (HFM), which achieves spatial resolution within a confined range; the second is a broad spatial-resolution mode powered by the Wolter mirrors, operating in a low-throughput configuration. Diagrams illustrating these two modes, as well as the configuration without the switching assembly, are shown in Figs. 3 ▸(*a*)–3(*c*).

In Mode 1, the addition of the HFM significantly enhances photon throughput. The surface parameters of the HFM are determined by the spatial position of the sample and detector and are independent of the dispersive subassembly. Considering that the spectrometer will be employed at an *in situ* endstation, the distance between the HFM edge and sample was set to 700 mm for installing the vacuum interlock protection system. At the same time, combined with the influence of the dispersive subassembly position on resolving power (see Section 2.3 for details), the HFM size was specified to be 325 mm × 40 mm. The full parameters of the HFM are listed in Table 1 ▸. Not only that, the spot size on the sCMOS detector in the non-dispersive direction can be adjusted to the <400 µm range by employing the focusing mirror, as shown in Fig. 3 ▸(*g*). In the current development of sCMOS detectors, nanostrip or microstrip detectors are rarely fabricated with a larger active area due to defects (Xiong *et al.*, 2022 ▸). Benefiting from the above single-dimensional small-sized spot, a strip-shaped detector can be designed and developed to minimize defects while maintaining the energy collection range in the dispersion direction. This approach not only reduces detector noise but also allows for the achievement of smaller pixels.

The photon throughput of the spectrometer is primarily influenced by three factors: collection angle, reflectivity of the optical components, and grating efficiency. Firstly, the HFM (collection mirror) in front of the dispersive subassembly has increased collection angles due to focusing of the emitted photons in the horizontal direction. A diagram illustrating the method for increasing the collection angle is shown in Fig. 3 ▸(*b*). If the collection mirror is not set up [without the HFM, as shown in Fig. 3 ▸(*a*)], the horizontal collection angle of the spectrometer is limited to 2.7 mrad due to the 13.3 mm active size of the detector; the beam spot size at the detector is simulated using *SHADOW* in Fig. 3 ▸(*g*). Moreover, the maximum collection angle can reach up to 10.6 mrad, limited by the size of the HFM (325 mm). Secondly, the reflectivity of the gold coating in the switching design is simulated using *XOP* (Sánchez del Rio & Roger, 2011 ▸). As shown in Fig. 3 ▸(*d*), the reflectivity of the HFM (blue line) significantly decreases after 1000 eV. Based on the results above, a comparison of total efficiencies considering three factors is displayed in Fig. 3 ▸(*f*). The HFM scheme increases the total efficiency by more than three times. The disparity gradually diminishes as energy increases, attributed to the influence of reflectivity. However, the advantages of the HFM are very prominent in the range below 1200 eV that we focus on (B/C/N/O/F *K*-edges, to 3*d* transition metal *L*-edges).

**Figure 4:**
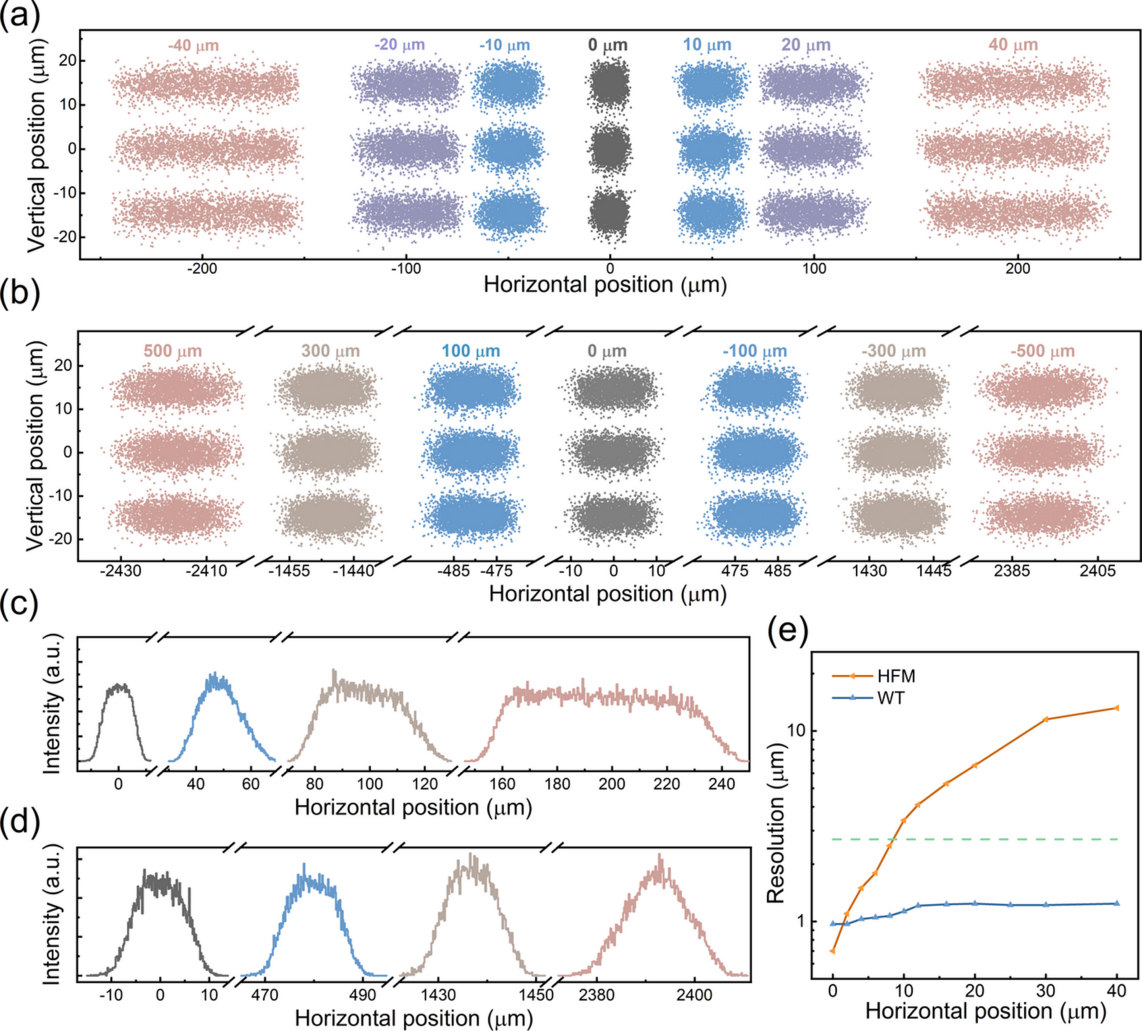
The spatial resolution performance of the spectrometer. (*a*) Images of the detector plane from −40 µm to +40 µm FOV at selected positions, with the HFM set in the optical path. The incident photon energies are selected to be 284 eV ± 23.7 meV. (*b*) Images of the detector plane from −500 µm to +500 µm FOV at selected positions, with the Wolter mirrors set in the optical path. The labels in each panel of (*a*) and (*b*) indicate the deviation of the source center position from the theoretical center position. (*c*) Histograms of the detector plane, with the HFM set in the optical path. (*d*) Histograms of the detector plane, with the Wolter mirrors set in the optical path. (*e*) The resolution limit of the Wolter mirror (blue line) and the HFM (orange line) as a function of horizontal position. The dashed line represents the highest resolution achievable under the current limitations of the detector.

Spatially resolved simulation results of the HFM scheme are shown in Fig. 4 ▸(*a*). Using the focusing relationship depicted in the layout, the magnification of the HFM is calculated to be 4.77. Additionally, the surface slope error for the elliptical cylindrical mirror is required to meet 0.2 µrad. Due to the independence of spatial imaging and energy resolution in the spectrometer, the photon energy value is uniformly set to 12000 @ 284 eV (LEG) at each position. At first, one can assert that energy resolution is unaffected by the movement of the source center based on the dispersion results in the *y*-direction. Meanwhile, the central position correspondence between source plane (sample) and image plane (CCD detector) is listed in Fig. S5(*a*). These results are in good agreement with the calculated magnification of HFM schemes. The spot width exhibits significant variation at different center positions. The FWHM of the image is given in the histograms in Fig. 4 ▸(*c*). After considering the magnification, the corresponding spatial resolutions can be calculated as 2.7 µm, 3.4 µm, 6.6 µm and 13.2 µm. To validate the reliability of the evaluation process, we also adjust the slit aperture based on the above calculated resolution; the image results are shown in Fig. S6.

## 4. Mode 2: Wolter-type mirrors

In this limited space by HFM, Wolter mirrors (WM1 and WM2) are added as an alternative providing horizontal imaging capability for spatial resolving power (Chuang *et al.*, 2020 ▸; Wolter, 1952*a* ▸; Wolter, 1952*b* ▸). The Wolter mirrors were inspired by the well established Wolter-type design (Chuang *et al.*, 2020 ▸; Warwick *et al.*, 2014 ▸). A hyperbolic cylindrical mirror (WM1) and an elliptical cylindrical mirror (WM2) are successively positioned downstream of the sample; the lengths of WM1 and WM2 have been limited by the HFM length to 130 mm and 140 mm, respectively. Not only that, but due to the included angle between the incident and reflected beams being limited to 176° by the HFM, the calculated common focus of WT1 and WT2 is located 1931.24 mm away from the center of WT1. It is worth noting that, although we have unified the included angle to 176°, due to the double reflection of the Wolter-type mirror, the parallel reflected beam paths in the two modes do not completely coincide but instead have a spacing of 0.17 mm. While this distance does not affect the position of downstream optical components, it will still result in a noticeable spot shift on the detector. The parameters of WM1 and WM2 are listed in Table 1 ▸. This provides users with the option of a wide spatial resolution range albeit at the cost of throughput. The selection will be determined based on the specific characteristics of the sample.

On the same principle, the angle of the Wolter mirrors is limited to 2.2 mrad. The footprints on the Wolter mirrors are shown in Fig. S7. A comparison of total efficiencies considering three factors is displayed in Fig. 3 ▸(*f*). The total efficiency using the Wolter mirrors scheme for spatial-resolution is only one-fifth of that of the HFM scheme.

Similar to the description of the HFM scheme, the spatially resolved simulation results and the center position correspondence are shown in Fig. 4 ▸(*b*), Fig. 4 ▸(*d*) and Fig. S5(*b*). Despite the significant drawbacks in terms of efficiency, the Wolter mirrors demonstrate excellent imaging characteristics. Clearly, the horizontal spot size (FWHM) remains largely unchanged across the beam spot range of ±40 µm at the sample. Furthermore, the resolution of 2.7 µm can be maintained throughout the full 600 µm field of view. Furthermore, the calculated resolution results are limited by the pixels of the CCD detector. If the detector could be updated to one with smaller pixels, the spectrometer would have better resolution. Calculated results of spatial resolution without considering the detector limitation are shown in Fig. 4 ▸(*e*). The limiting resolution of the Wolter mirrors is approximately 1 µm (blue line) and the FOV can be extended to 0.25 mm. Compared with the current resolution (dashed line), upgrading the detector is the most effective method to enhance resolution when compared with other approaches, such as lengthening the spectrometer or improving the precision of the optical components. However, the resolution of the HFM exceeds the detector limit at the central position of 8 µm, making it difficult to improve further. The calculation method for limiting resolution is provided in Section S3.

## 5. Simultaneous spatial and energy resolution: interdependent errors

**Figure 5:**
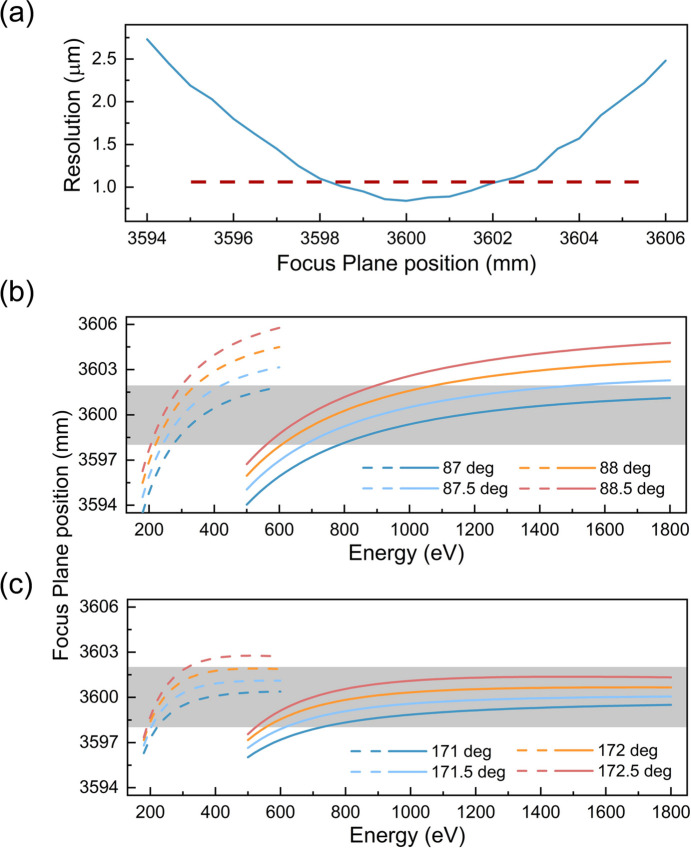
Calculation of the focal plane position. (*a*) Spatial resolution at different focal plane positions (central position at 3600 mm). (*b*) Variation of the focal plane positions with energy under a fixed *b*_2_ for the constant incident angle method. (*c*) Variation of focal plane positions with energy under a fixed *b*_2_ for the constant included angle method.

To achieve simultaneous focusing in both the horizontal and vertical directions, the HU structure was selected due to its fixed focal plane in terms of dispersion characteristics (Dvorak *et al.*, 2016 ▸). However, the focal plane is not completely fixed when the detail of the HU structure is further considered by the focusing equation. If the movement of the detector is employed to ensure optimal energy resolution, it will inevitably lead to a degradation in spatial resolution in the horizontal direction. Therefore, evaluating the impact of offset on spatial resolution is also an important factor in assessing the performance of the spectrometer. Firstly, the variation of spatial resolution with the position of the focal plane is calculated, as shown in Fig. 5 ▸(*a*). For the calculated maximum spatial resolution of 1 µm, the change in the focal plane should be less than ±1.6 mm. Based on this, and in accordance with Section 2, the moment requirements of the detector (*r*_2_) are calculated as follows,
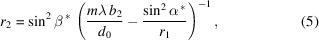
where *b*_2_ has been fixed by a fabricated grating. The calculation results for the two methods of constant incident angle and constant included angle are shown in Figs. 5 ▸(*b*) and 5 ▸(*c*), where the range of spatial resolution limitations is indicated by the gray shaded areas. It can be seen that, in the case of a single incident angle or a single included angle, the position of the focal plane cannot be maintained within ±1.6 mm. When adjusting the angle within a range of 1.5° in the figure, the aforementioned issue can be corrected relatively well. However, it is still difficult to correct the low-energy region (180–210 eV). Further increases in the angle may introduce other errors (such as *a*_2_) affecting resolution, and limit the collection angle thereby decreasing efficiency.

## 6. Determining mechanical tolerances

In the aforementioned spectrometer design, there are high-precision focusing requirements in two dimensions. Consequently, the installation accuracy and stability of the optical components are crucial for achieving optimal imaging results. As a movable spectrometer, the relative positioning accuracy of optical components serves as a key metric that mechanical designers must consider. Herein, geometry calculations and *SHADOW* simulations are combined to evaluate the influence of optical misalignment across different dimensions on both horizontal and vertical imaging quality. To establish the installation accuracy, distinct tolerance criteria have been established for various components. Firstly, a horizontal spectral tail on the detector plane not exceeding 5 µm is used to evaluate the tolerances of the HFM. In contrast, the standard for the Wolter mirrors has been tightened to 1 µm to accommodate potential upgrades to the detector. Additionally, evaluation of the main components for energy resolution also considers the potential upgrade of the detector. The final requirement is that the vertical spectral tail should not exceed 2 µm. The tolerances are allocated to each optical component in a non-uniform manner based on practical considerations. The final results are summarized in the fourth column of Table 2 ▸ ▸. Meanwhile, the *SHADOW* simulation results of Mθ_*X*_ and MZ tolerances are shown in Table S2.

In addition to installation accuracy, the stability of the components during the testing process is also a critical requirement that affects the test results (Dvorak *et al.*, 2016 ▸). Here, calculations are made based on the standard that the offset should not exceed 3 µm (RMS, 25% effective pixel resolution). Since the requirements of the HFM are more relaxed compared with those of Wolter mirrors and the same mechanism is used for design convenience, the parameters of the HFM are based on those of Wolter mirrors. The calculated results are summarized in the fifth column of Table 2 ▸. However, the stability can be influenced by many factors such as site-wide and local vibration sources (Spataro *et al.*, 2018 ▸). The stability given is a reference for the mechanical designer to verify the feasibility of the design, and then modify it appropriately.

Moreover, source point motion (*i.e.* photon position on the sample) critically impacts the imaging results. This is determined by multiple factors, including the system-level alignment accuracy of the spectrometer, the precision of the sample stage, and the focal spot position of the beamline. Herein, we simplify the error analysis by focusing on source motion in three spatial directions. Among these, horizontal source motion has been comprehensively shown in Fig. 4 ▸. The analysis for the other two directions is summarized in Table S3. Based on the results, the tolerance for vertical source motion is 30 µm, while displacements along the optical path are constrained to 300 µm.

## 7. Conclusion and outlook

We have presented an optical design for a RIXS spectrometer at Hefei Advanced Light Facility. This design incorporates a switching assembly positioned in front of the existing VLS–HU design, allowing for a switch between two operational modes: high throughput mode (utilizing an elliptical cylindrical mirror) and spatial-resolved mode (utilizing a combination of a hyperbolic cylindrical mirror and an elliptical cylindrical mirror).

The VLS–HU design configuration with two gratings covers an energy range of 180–1800 eV and achieves a high resolving power (exceeding 12000) for two grating rotation methods: constant incident angle and constant included angle. The switching assembly in front of the VLS–HU HFM simultaneously fulfills three functions: (i) increases throughput, (ii) adds spatial resolution functionality and (iii) provides a focusing mode to reduce noise and offer potential for upgrading the detector. In the spectrometer, with a total length of only 5 m, the spatial resolution at the central position can reach 2.7 µm. An alternative option of using Wolter mirrors offers a broad field of spatial resolution, achieving a resolution of 2.7 µm within an 80 µm range. However, the drawback is that the photon throughput of Wolter mirrors is only 20% of the HFM.

Furthermore, regarding the design of the aforementioned multi-optical elements, the mechanical tolerances have been further evaluated in order to facilitate the ongoing mechanical design work. However, these efforts are insufficient for the subsequent stages. The design of the mirror tank is a key focus in the construction of the spectrometer, aiming to achieve high-precision optical functionality that enables simultaneous collection of spatial and energy information, while also considering convenience and high stability during usage. These efforts will significantly influence the user experience of this spectrometer in the future.

## Supplementary Material

Supporting information. DOI: 10.1107/S1600577525005314/vy5038sup1.pdf

## Figures and Tables

**Figure 6:**
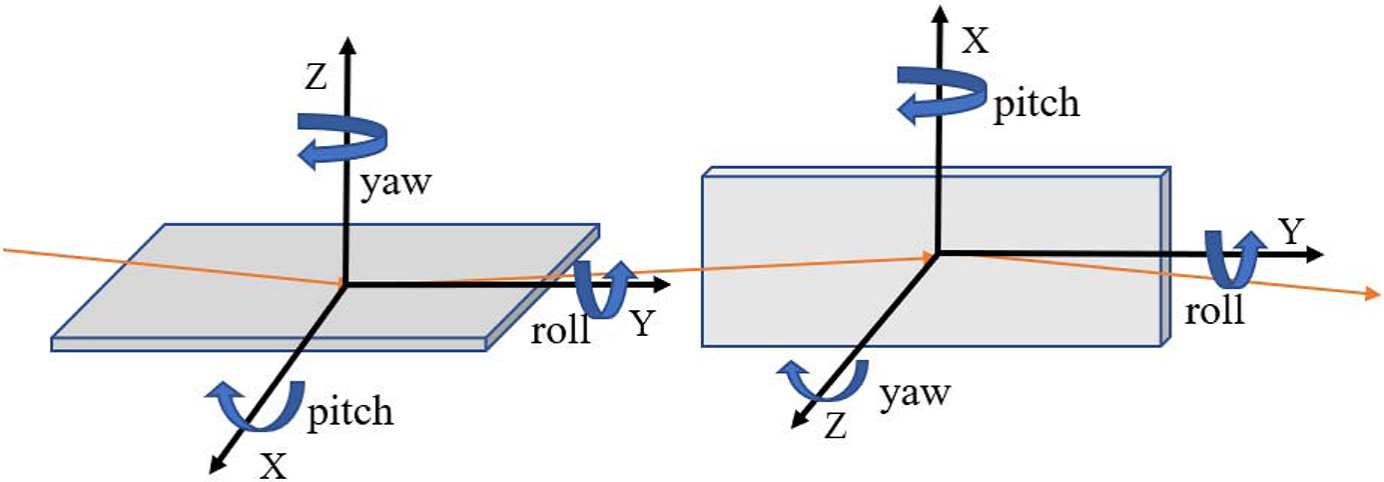
Schematic of the coordinate system.

**Table:** 

	Shape of optical surface	Dimensions of mirror (L × W × T) (mm)	Incident angle (θ)	Slope error (µrad)		Entrance arm Exit arm (mm)		Semi-major Semi-minor (mm)	
HFM	Elliptical cylindrical	325 × 40 × 30	88°	0.2 µrad		862.5		2495.0	
						4127.5		65.85	
EM	Elliptical cylindrical	180 × 40 × 30	88°	0.2 µrad		1210		2495.0	
						3780		74.64	
WM1	Hyperbolic cylindrical	130 × 40 × 30	89°	0.2 µrad		1931.24		583.12	
						765.0		21.21	
WM2	Elliptical cylindrical	140 × 40 × 30	89°	0.2 µrad		2121.24		3078.12	
						4035.0		51.06	

**Table:** 

	Shape of optical surface	Active energy range (eV)	Dimensions of grating (L × W × T) (mm)		Slope error (µrad)		VLS terms		Error bars for VLS terms
LEG	Plane	180–600	180 × 40 × 40		0.2 µrad		*N*_0_ = 1200 lines mm^−1^		*b*_2_: ±0.2%
							*b*_2_ = 0.6644 lines mm^−2^		
							*b*_3_ = 2.75 × 10^−4^ lines mm^−3^		
							*b*_4_ = 2.2 × 10^−7^ lines mm^−4^		
HEG	Plane	600–1800	180 × 40 × 40		0.2 µrad		*N*_0_ = 2800 lines mm^−1^		*b*_3_: ±10%
							*b*_2_ = 1.5511 lines mm^−2^		
							*b*_3_ = 6.41 × 10^−4^ lines mm^−3^		
							*b*_4_ = 2.3 × 10^−7^ lines mm^−4^		

**Table 2:** Alignment tolerance and stability of mechanical motion The third column indicates the direction of influence on imaging. A schematic of the coordinate system is shown in Fig. 6 ▸.

		Imaging direction	Alignment tolerance	Stability
HFM	MX (transverse)	N/A	1 mm	N/A
	MY (along beam path)	Horizontal	20 µm	N/A
	MZ (up/down)	Horizontal	5 µm	N/A
	Mθ_*X*_ (pitch)	Horizontal	17 µrad	N/A
	Mθ_*Y*_ (roll)	Vertical	175 µrad	N/A
	Mθ_Z_ (yaw)	Horizontal and vertical	1.7 mrad	N/A

EM	MX (transverse)	N/A	1 mm	N/A
	MY (along beam path)	Vertical	50 µm	±0.5 µm
	MZ (up/down)	Vertical	30 µm	±0.1 µm
	Mθ_X_ (pitch)	Vertical	17 µrad	±0.69 µrad
	Mθ_Y_ (roll)	Horizontal	100 µrad	±2 µrad
	Mθ_Z_ (yaw)	Horizontal and vertical	1.7 mrad	N/A

WM1	MX (transverse)	N/A	1 mm	N/A
	MY (along beam path)	Horizontal	100 µm	±0.5 µm
	MZ (up/down)	Horizontal	13 µm	±0.1 µm
	Mθ_X_ (pitch)	Horizontal	7 µrad	±0.29 µrad
	Mθ_Y_ (roll)	Vertical	175 µrad	N/A
	Mθ_Z_ (yaw)	Horizontal and vertical	9 mrad	N/A

WM2	MX (transverse)	N/A	1 mm	N/A
	MY (along beam path)	Horizontal	300 µm	±0.5 µm
	MZ (up/down)	Horizontal	20 µm	±0.1 µm
	Mθ_X_ (pitch)	Horizontal	9 µrad	±0.29 µrad
	Mθ_Y_ (roll)	Vertical	90 µrad	N/A
	Mθ_Z_ (yaw)	Horizontal and vertical	9 mrad	N/A

LEG and HEG	MX (transverse)	N/A	1 mm	N/A
	MY (along beam path)	Vertical	500 µm	±0.5 µm
	MZ (up/down)	Vertical	70 µm	±0.1 µm
	Mθ_X_ (pitch)	Vertical	1.0 µrad	±0.6 µrad
	Mθ_Y_ (roll)	Horizontal	1.7 mrad	N/A
	Mθ_Z_ (yaw)	Horizontal and vertical	1 mrad	N/A
