# Supplementary material for: A high throughput and micrometre spatial resolution design for a versatile resonant inelastic X-ray scattering spectrometer
Source: J Synchrotron Radiat. 2025 Jul 17;32(Pt 5):1235–43. doi: 10.1107/S1600577525005314 (PMC12416433; doi:10.1107/S1600577525005314)
Supplement: Supplementary file 1 [file s-32-01235-sup1.pdf]

JOURNAL OF
SYNCHROTRON
RADIATION

**Volume 32 (2025)**

**Supporting information for article:**

# A high throughput and micrometre spatial resolution design for a versatile resonant inelastic X-ray scattering spectrometer

**Ruijie Wang, Pengjun Zhang, Qingchen Li, Yujian Xia, Lisheng Qian, Xuefei Feng, Shuangming Chen and Xiaosong Liu**

### S1. Optical scheme of beamline

The beamline is a soft X-ray spectroscopy and scattering station at Hefei Advanced Light Facility. The energy range for the beamline will be provided from 180 eV to 2500 eV. The layout of the beamline is presented in Fig.S1. Unlike the design of conventional Kirkpatrick–Baez (KB) mirrors in beamline, the elliptical cylinder mirror (M5) in the branch line is positioned independently at 42 m. It serves as the horizontal focusing mirror within the KB configuration and is also used to switch from main beamline (NAP-XPS) to branch beamline (RIXS). Benefiting from the significant difference in focus ratio between M5 and M6, the beam spot size is approximately 58 μm × 2.5 μm (H × V) at 244 eV.

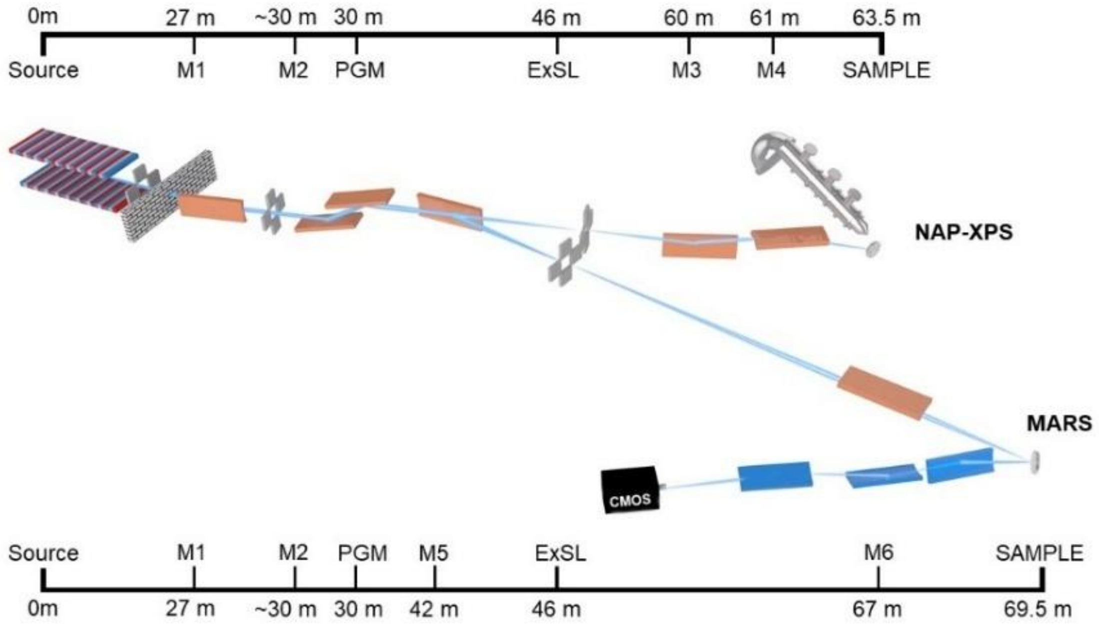


**Figure S1** The layout of the beamline.

## S2. Energy resolution of spectrometer

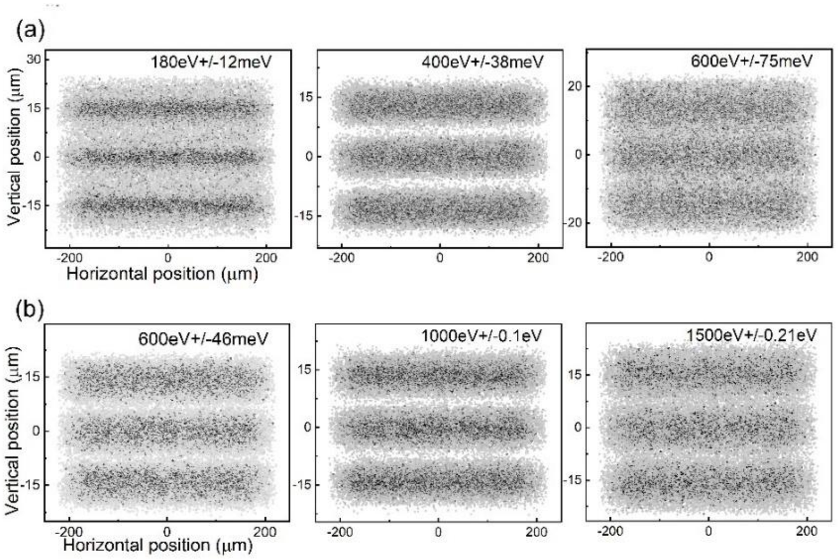


**Figure S2** The SHADOW simulations of the spectrometer with constants incident angle: (a) the SHADOW simulations at detector plane at 180 eV, 400 eV and 600 eV (using LEG). (b) the SHADOW simulations at detector plane at 600 eV, 1000 eV, and 1500 eV (using HEG). The detector needs to be moved 4 mm forward at 180 eV. In the low energy range, the constants incident angle method is not used due to excessively large incident angle.

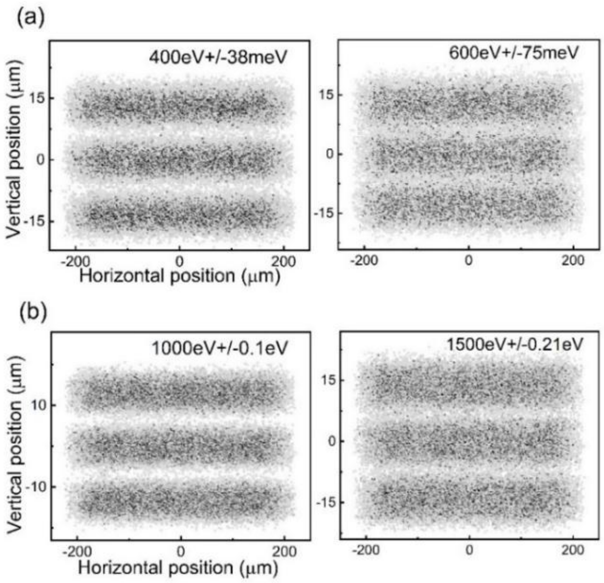


**Figure S3** The SHADOW simulations of the spectrometer with constants include angle method: (a) the SHADOW simulations at detector plane at 180 eV, 400 eV and 600 eV (using LEG). (b) the SHADOW simulations at detector plane at 600 eV, 1000 eV, and 1500 eV (using HEG).

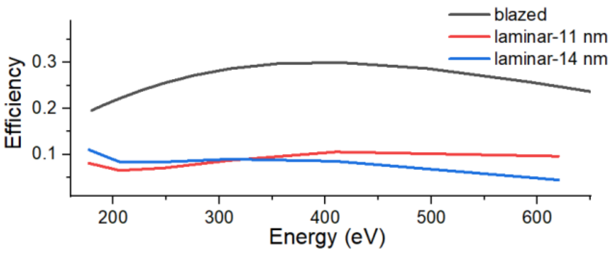


**Figure S4** The laminar grating efficiency at 0.3 duty cycle with groove depths of 11 nm and 14 nm, taking the LEG as an example.

### S2.1. The effect of errors in VLS terms

As mentioned in Table 1, the errors in VLS terms are considered the impact on the spectrometer and the adjustment methods. Firstly, regarding the 0.2% error in the $b_2$ term, our evaluation has shown that adjusting the grating angle have a minimal effect on mitigating the error. Consequently, we plan to adjust the grating center along the optical path direction (2 mm) and combine this with the adjustment of the detector position (1 mm) to achieve optimal error elimination. Then, the situation with $b_3$ term is different, adjusting the grating angle demonstrates a greater impact. The final evaluation indicates that the incident angle requires an adjustment of 0.4 deg.

Taking 292 eV as an example, the Table S1 below compares the simulation results before and after the adjustment. Although the correction cannot fully offset the impact of errors due to the detector pixel being the primary limiting factor for the energy resolving power of spectrometer, the overall resolving power (12,000 @ 292 eV) remains unchanged. The current assessment is a first step before the mechanical design. Upon completion of grating fabrication, the optical layout will be fine-tuned using actual measurements of the grating and other optics.

**Table S1** Correction results for the errors in VLS term by adjusting grating center along the optical path ($b_2$) or adjusting the incident angle ($b_3$).

| | $b_2$ | $b_3$ |
|---|---|---|
| Before adjustment | 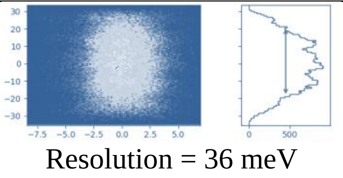 Resolution = 36 meV | 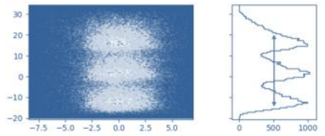 Resolution = 24.5 meV |
| After adjustment | 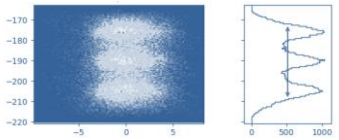 Resolution = 23.7 meV | 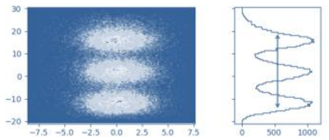 Resolution = 23.5 meV |

## S3. Spatial resolution of spectrometer

### S3.1. Calculation Method for Spatial Resolution

The realization of spatial resolution is based on the magnification imaging of sample points by the spectrometer. Therefore, the calculation method for spatial resolution (SRP) is as follows:

$$SRP = Wide / M,$$

Wide is the minimum recognizable size on the detector, and M is the magnification ratio. In this design, the switching assembly provides a magnification ratio of 4.78. Then the minimum recognizable size on the detector is determined by two factors: (i) the effective pixel size (wide1) of the detector, (ii) the minimum focused spot size (wide2) influenced by the slope error of the HFM or WM. Firstly, the former is the primary limiting factor for spatial resolution in this work, and the corresponding calculated spatial resolution is 2.7 μm (13 μm / 4.77). When the detector limitations are not considered, the calculation method for wide2 as the primary factor is as follows:

$$wide2 = 4.7\frac{slope1 * L1}{M2} + 4.7\, slope2 * L2,$$

In the above, slope1 and slope2 are the slope errors of the two mirrors in the switching assembly, for Mode1 which only includes one mirror only the slope2 is considered. L1 and L2 are the exit arm lengths corresponding to the focusing surface type, M2 is the magnification ratio of the second mirror. For calculations involving off-center parts, specific values are also combined with ray-tracing results.

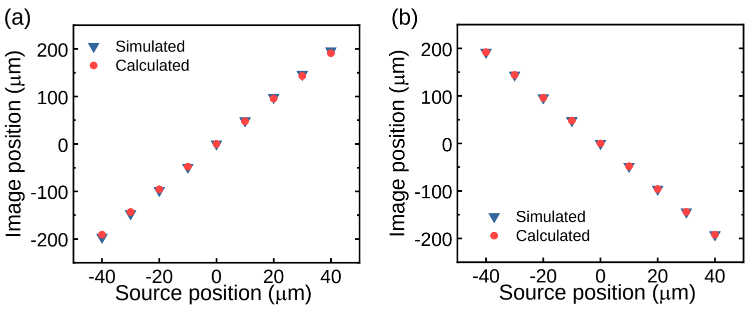


**Figure S5** Correspondence between simulation (blue points) and calculation (red points) values of central. (a) HFM design. (b) Wolter mirrors design.

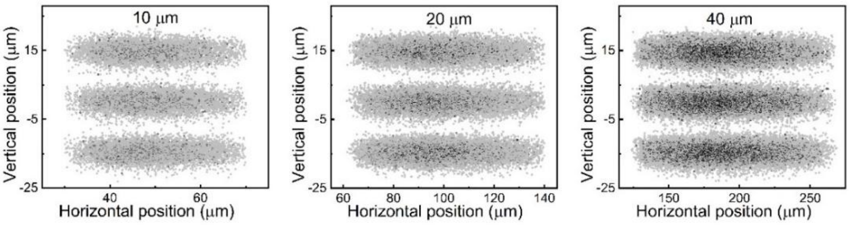


**Figure S6** The SHADOW simulations of the spectrometer with different source center position when the slit aperture positioned downstream of the source is adjusted based on the calculated spatial resolution. The titles in each panel indicate the deviation of the source center position from the theoretical center position.

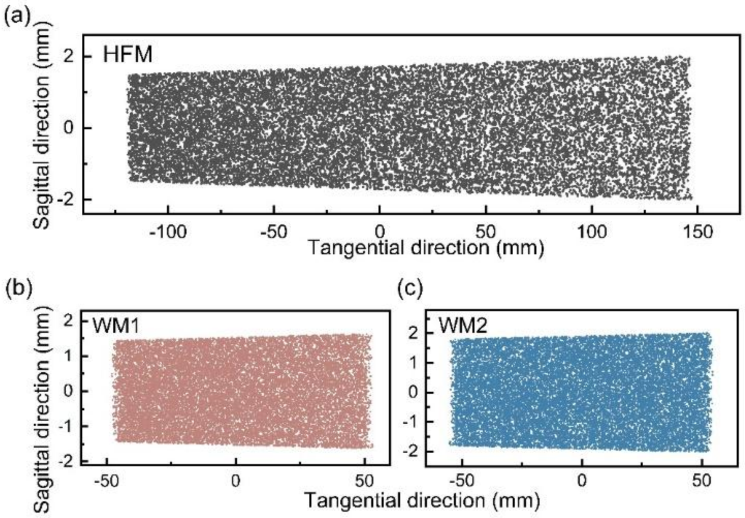


**Figure S7** Footprints of HFM and Wolter mirrors. (a) The footprint of HFM, the one sigma value is 75.91 mm. (b) The footprint of WM1, the one sigma value is 27.84 mm. (c) The footprint of WM2, the one sigma value is 30.51 mm.

## S4. Mechanical tolerances

**Table S2** The SHADOW simulation results of key dimensional tolerances ($M\theta_X$, MZ) for HFM, EM, and PG. The results of WM1 and WM2 (consistent with HFM) are omitted. The $M\theta_X$ tolerance of HFM significantly impacts spot size, but z-axis correction (15 µm) resolves this. The table presents the corrected results. The caption below describes the size of the spot shown in the diagram, with the initial size being 12.33 µm × 5.27 µm.

| | $M\theta_X$ (pitch) | MZ |
|---|---|---|
| HFM | 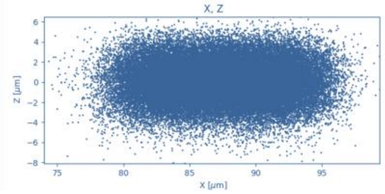<br>11.72 µm × 5.05 µm | 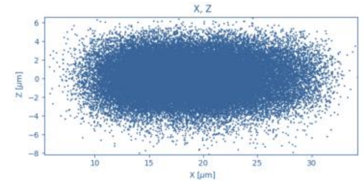<br>12.05 µm × 5.27 µm |
| EM | 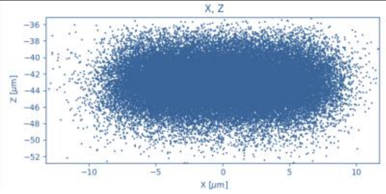<br>12.58 µm × 5.30 µm | 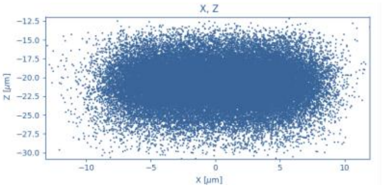<br>12.13 µm × 5.68 µm |
| PG | 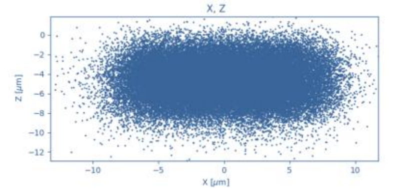<br>12.52 µm × 5.16 µm | 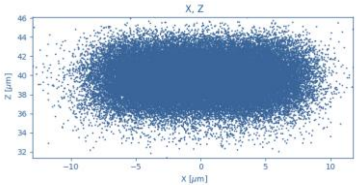<br>12.19 µm × 5.19 µm |

**Table S3** The SHADOW simulation results of the impact of source point motion. The source center position is adjusted by incorporating an addition slit. The resolution calculated based on the 13 μm effective pixel of the detector.

| Vertical source motion | | | |
|---|---|---|---|
| Motion | 0 μm | ±30 μm | ±50 μm |
| | 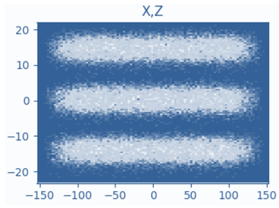  | 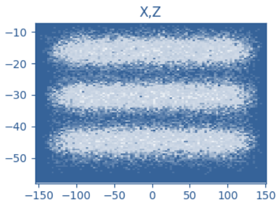  | 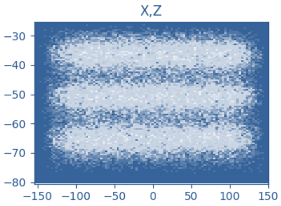  |
| Resolution | 21.6 meV @284 eV | 21.5 meV @284 eV | 22.0 meV @284 eV |
| Source motion along the optical path | | | |
| Motion | 0 μm | ±300 μm | ±500 μm |
| | 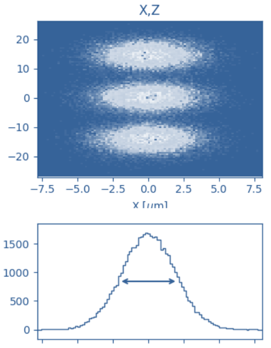  | 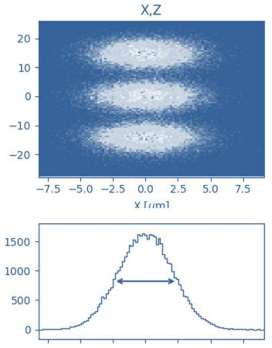  | 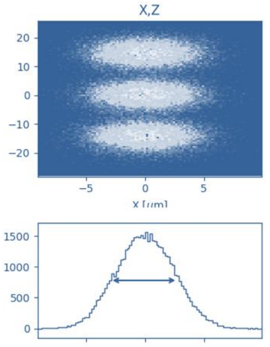  |
| Energy resolution | 21.6 meV @284 eV | 21.8 meV @284 eV | 21.8 meV @284 eV |
| Spatial resolution | 0.86 μm | 1.02 μm | 1.18 μm |
